# Diagnostic Accuracy of Point-of-Care Ultrasound (POCUS) for Suspected Acute Appendicitis in Pediatric and Adult Emergency Departments: A Systematic Review and Meta-Analysis

**DOI:** 10.7759/cureus.103503

**Published:** 2026-02-12

**Authors:** Mohamed M Kamal, Bandar M Al Reshidi, Haytham M Bedier, Abdullah O Albariqi, Wateen A Almotrafie, Bandar S Alshreef, Mohammed O Wahbi, Waseema Aly, Saleh A Alyami, Hessah Abdallah, Shahad A Alharbi

**Affiliations:** 1 Radiodiagnosis, King Khalid Hospital, Hail, SAU; 2 Radiology, Saudi Arabia Ministry of Health, Hail, SAU; 3 Radiology, Afif General Hospital, Riyadh Third Cluster, Afif, SAU; 4 Diagnostic Radiology, General Directorate Prisons Health, Jeddah, SAU; 5 Pharmacy, College of Pharmacy, Northern Border University, Arar, SAU; 6 Healthcare Management, College of Applied Medical Sciences, Shaqra University, Shaqra, SAU; 7 Diagnostic Radiology, Bedfordshire Hospitals NHS Foundation Trust, Bedford, GBR; 8 Diagnostic and Interventional Radiology, Barts NHS Health Trust, London, GBR; 9 Diagnostic Radiology, King Saud Medical City, Riyadh, SAU; 10 Diagnostic Radiography, King Faisal Medical Complex, Taif, SAU; 11 Medicine and Surgery, Taibah University, Madinah, SAU

**Keywords:** acute appendicitis, diagnostic accuracy, emergency medicine, meta-analysis, pocus, point-of-care ultrasound

## Abstract

Acute appendicitis is a common surgical emergency that often requires diagnostic imaging. While computed tomography (CT) is highly accurate, it involves ionizing radiation and potential delays. Point-of-care ultrasound (POCUS), performed by emergency physicians, offers a rapid, radiation-free alternative, but its diagnostic accuracy varies across studies. This systematic review and meta-analysis evaluates the diagnostic performance of POCUS for acute appendicitis in pediatric and adult populations. Major databases were searched for prospective and retrospective studies evaluating POCUS for appendicitis performed by emergency physicians. The primary outcomes were sensitivity, specificity, and likelihood ratios. Quality assessment was performed using the Quality Assessment of Diagnostic Accuracy Studies-2 (QUADAS-2) tool. Data were synthesized using a bivariate random-effects model, and a Hierarchical Summary Receiver Operating Characteristic (HSROC) curve was generated. Subgroup analyses explored heterogeneity based on patient population and operator experience. Fifteen studies involving 2,021 patients were included. POCUS demonstrated a pooled sensitivity of 0.78 (95% CI: 0.69-0.85) and a pooled specificity of 0.89 (95% CI: 0.83-0.93). The positive likelihood ratio was 7.22 (95% CI: 4.51-11.54), and the negative likelihood ratio was 0.25 (95% CI: 0.17-0.36). Significant heterogeneity was observed, partly explained by operator experience, with fellowship-trained physicians achieving higher accuracy than residents. Publication bias was detected via Deeks’ funnel plot asymmetry test (P < 0.01). POCUS performed by emergency physicians is a highly specific and moderately sensitive tool for diagnosing acute appendicitis. A positive scan reliably rules in the disease, potentially reducing the need for further imaging. However, a negative scan is insufficient to rule out appendicitis, requiring secondary evaluation. Structured training and experience are critical for optimizing diagnostic performance.

## Introduction and background

Acute appendicitis ranks among the most common conditions requiring urgent surgical intervention, carrying an estimated lifetime risk of 6.7% for females and 8.6% for males [1,2]. Although it is a primary reason for abdominal surgery in patients of all ages, accurately diagnosing the condition continues to present substantial clinical difficulties [3]. A delayed diagnosis increases the risk of complications, such as perforation and abscess formation, which are associated with significant morbidity [4]. Misdiagnosis may lead to negative appendectomies, subjecting patients to unnecessary anesthesia and surgical risks [2,3].

Computed tomography (CT) is the gold standard imaging modality because of its high sensitivity (94-100%) and specificity (93-100%) [5]. However, the use of CT is tempered by concerns regarding ionizing radiation exposure, particularly in the pediatric population, where it is associated with a projected increase in lifetime cancer risk [5,6]. Ultrasound (US) is recommended by major medical societies as the first-line imaging modality for children and young adults [2,6]. While a formal radiology department ultrasound (RUS) avoids radiation and offers high specificity, it is often limited by the availability of sonographers and radiologists, particularly during off-hours, potentially prolonging the emergency department (ED) length of stay and delaying definitive management [4,6].

To address these logistical limitations, point-of-care ultrasound (POCUS) performed by emergency physicians has emerged as a valuable bedside diagnostic tool [7]. POCUS facilitates rapid, non-invasive, and real-time assessment of the appendix using graded compression techniques to identify key pathological signs, such as a non-compressible, blind-ending tubular structure with a diameter exceeding 6 mm [8,9]. Recent data suggest that POCUS may offer diagnostic accuracy comparable to that of RUS, with potential benefits including reduced healthcare costs and expedited time to disposition [6,7].

Despite its utility, the diagnostic performance of POCUS is operator-dependent and may be influenced by patient-specific factors [3,7] as diagnostic accuracy is compromised in patients with a high body mass index (BMI) or those with a low pre-test clinical probability of appendicitis, leading to non-diagnostic scans [10]. Furthermore, while some meta-analyses suggest that POCUS demonstrates high specificity (up to 91-97%), its sensitivity varies significantly across studies (72-94%), often necessitating secondary imaging, such as CT or MRI, to rule out pathology definitively [4,6,7]. The high heterogeneity among existing studies regarding operator experience and patient demographics further complicates the establishment of universal performance benchmarks [3,7].

Given the variance in reported accuracy and the critical need to balance diagnostic precision with resource stewardship and radiation safety, a consolidated evaluation of the current evidence is warranted. The objective of this systematic review and meta-analysis was to assess how accurately emergency physicians can diagnose suspected acute appendicitis using POCUS in pediatric and adult cohorts, specifically measuring its effectiveness as a tool to rule in or rule out the disease in emergency settings.

## Review

Methods

Protocol and Registration

This systematic review and meta-analysis was conducted in accordance with the Preferred Reporting Items for Systematic Reviews and Meta-Analyses (PRISMA) guidelines [10], and the protocol was registered with the International Prospective Register of Systematic Reviews (PROSPERO; CRD420251167771).

Search Strategy

A systematic literature retrieval was executed across primary electronic repositories, including Ovid MEDLINE, Embase, Cochrane Central Register of Controlled Trials (CENTRAL), and Google Scholar, from their inception through December 2025. The search protocol was designed in consultation with a medical librarian, employing a syntax of Medical Subject Headings (MeSH) and free-text terms such as "bedside ultrasound", "emergency sonography", and "acute abdomen". The reference lists of the included studies and relevant systematic reviews were manually screened to identify additional eligible articles. While no language filters were applied during the initial literature search, only studies with full texts available in English were included in the final synthesis to ensure accurate data extraction.

Inclusion and Exclusion Criteria

Studies were eligible for inclusion if they were prospective or retrospective observational studies or randomized controlled trials (RCTs) evaluating adult or pediatric patients presenting to the emergency department with suspected acute appendicitis. To meet the criteria, the index test must have been POCUS performed at the bedside by emergency physicians, including residents or attendings, compared with a reference standard of surgical pathology, clinical follow-up, or advanced imaging such as CT or MRI. Additionally, studies were only included if they contained adequate data to allow for the extraction of true positives, false positives, true negatives, and false negatives to populate a 2 × 2 diagnostic table.

Studies in which ultrasound was performed by radiologists or technicians, as well as case reports, editorials, reviews, and conference abstracts lacking full data, were excluded. Furthermore, studies focusing solely on pregnant women were excluded unless separate data for the general population were available or the study specifically aimed to validate POCUS in pregnancy, as were any articles containing insufficient data to calculate sensitivity and specificity.

Methodological Quality and Risk of Bias Assessment

The methodological rigor of the selected articles was evaluated independently by two reviewers employing the Quality Assessment of Diagnostic Accuracy Studies-2 (QUADAS-2) instrument [11]. This tool appraises the risk of bias across four specific domains: patient selection, index test execution, reference standard validity, and flow/timing. To ensure comprehensive reporting transparency within the primary literature, adherence to reporting standards was evaluated using the Standards for Reporting Diagnostic Accuracy 2015 (STARD 2015) checklist [12]. Discrepancies in the quality assessment were resolved through consultation with a third reviewer.

Statistical Analysis and Modeling

All statistical analyses were performed using R software (version 4.5.1; R Foundation for Statistical Computing, Vienna, Austria) [13]. As sensitivity and specificity are not independent variables in diagnostic accuracy studies, a bivariate random-effects model was employed to calculate the pooled estimates of sensitivity and specificity with 95% confidence intervals (CIs) [14]. To visually assess the trade-off between sensitivity and specificity across studies and to estimate the summary receiver operating characteristic curve, a Hierarchical Summary Receiver Operating Characteristic (HSROC) model was constructed [15].

The effect measures calculated included the positive likelihood ratio (LR+), negative likelihood ratio (LR-), and diagnostic odds ratio (DOR). For studies reporting agreement between bedside POCUS and expert retrospective review, inter-rater reliability was synthesized using Cohen’s kappa statistics [16]. To demonstrate clinical utility, Fagan’s nomogram was generated to calculate the post-test probabilities of acute appendicitis based on relevant pre-test probability thresholds [17].

Investigation of Heterogeneity and Robustness

Statistical heterogeneity (inconsistency) was assessed using the I2 statistic and visual inspection of the coupled forest plots [18]. The 95% prediction intervals were calculated to estimate the range in which the true diagnostic effect of a future study is expected to lie, providing a measure of dispersion beyond the confidence interval [19].

To explore the potential sources of heterogeneity, moderators were analysed using subgroup analysis and meta-regression. The prespecified subgroups included patient population (pediatric vs. adult), operator experience (resident vs. fellowship-trained/attending), and body mass index (BMI). The robustness of the results was further tested through sensitivity analyses, excluding studies with a high risk of bias.

Assessment of Bias

Reporting and dissemination biases, particularly small-study effects, were evaluated using a visual assessment of bias via funnel plots. Statistical tests for small-study effects were conducted using Deeks’ funnel plot asymmetry test, which is specifically adapted for diagnostic test accuracy meta-analyses to avoid the high false-positive rates associated with standard regression tests [20].

Certainty of Evidence

The overall certainty and strength of the body of evidence were assessed using the Grading of Recommendations Assessment, Development, and Evaluation (GRADE) approach [21]. Evidence was graded as high, moderate, low, or very low quality based on the risk of bias, indirectness, inconsistency, imprecision, and publication bias.

Results

Study Selection and Characteristics

**Figure 1:**
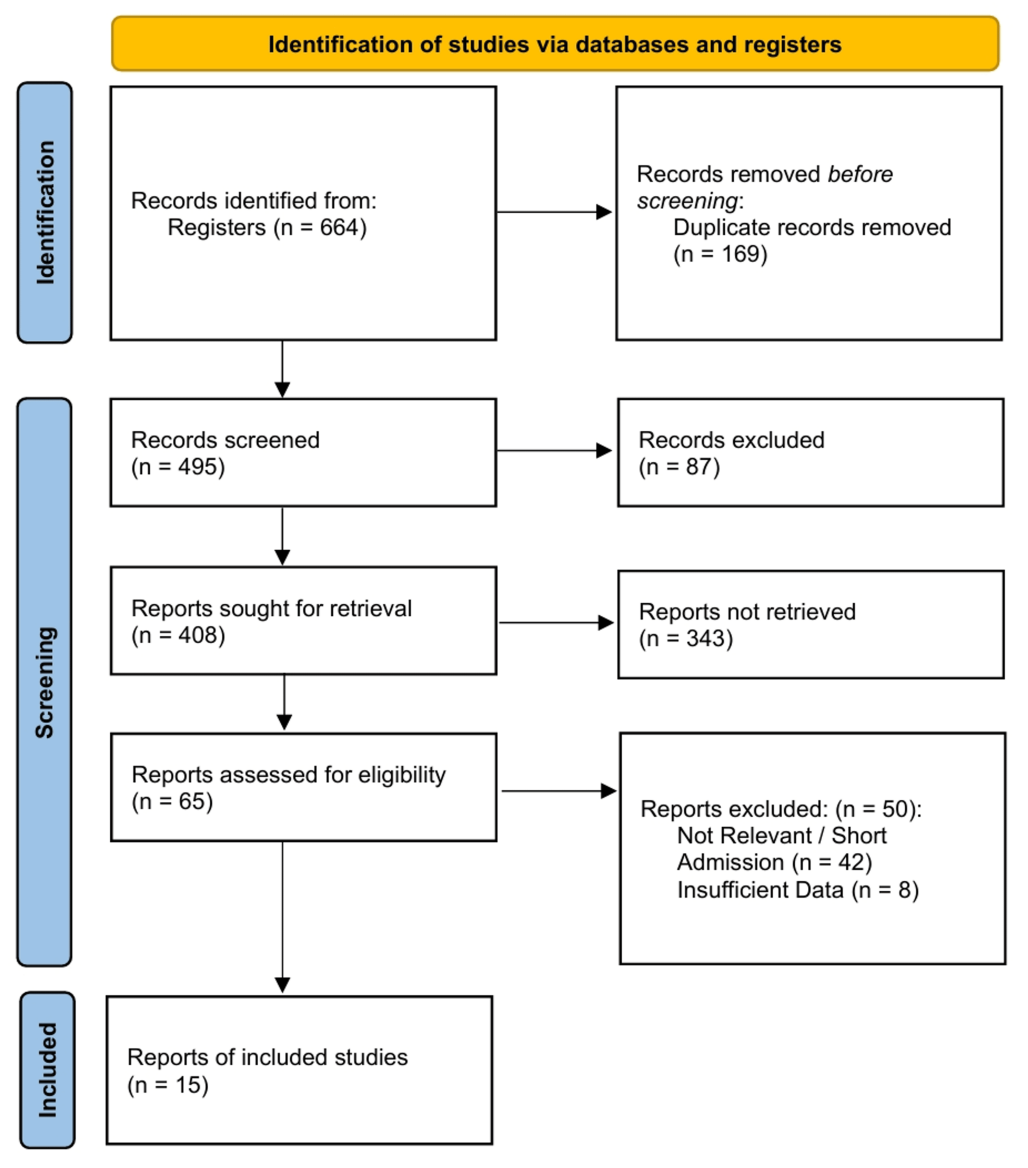
PRISMA 2020 Flow Diagram Abbreviations: PRISMA, Preferred Reporting Items for Systematic Reviews and Meta-Analyses

The search strategy identified 664 records from electronic databases and registers. After eliminating 169 duplicate entries, we screened 495 titles and abstracts, eventually retrieving 408 records. Sixty-five full-text articles were assessed for eligibility using the STARD 2015 criteria [12]. Fifty studies were excluded due to non-relevant comparator groups (n=42 admission-based studies) or insufficient data to construct 2 × 2 contingency tables (n=8). Fifteen studies meeting the inclusion criteria were included in the quantitative synthesis [22-36]. The selection process is detailed in the PRISMA flow diagram in Figure 1.

The meta-analysis comprised a total cohort of 2,021 patients from pediatric, adult, and mixed populations. The prevalence of acute appendicitis within the study populations varied from 28.1% to 64%. Operator experience varied significantly, ranging from residents with limited training to fellowship-trained ultrasound faculty and attending physicians (Table 1).

**Table 1:** Characteristics of the Included Studies Abbreviations: N, sample size; POCUS, point-of-care ultrasound.

Reference No.	Author (Year)	Country	Design	Population	N	Prevalence (%)	Operator	Reference Standard
[22]	Becker et al. (2022)	USA	Prospective	Mixed	256	28.1	Attending/Fellow/Resident	Pathology/Follow-up
[23]	Doniger & Kornblith (2016)	USA	Prospective	Pediatric	40	40.0	Fellow/Resident	Pathology/Follow-up
[24]	Elikashvili et al. (2014)	USA	Prospective	Pediatric	150	33.3	Fellow/Attending	Pathology/Follow-up
[25]	Fox et al. (2008)	USA	Prospective	Mixed	132	33.3	Attending/Resident	Pathology/Follow-up
[26]	Lam et al. (2014)	USA	Prospective	Mixed	116	40.0	Attending	Pathology/Follow-up
[27]	Chen et al. (2000)	Taiwan	Prospective	Mixed	147	74.8	Attending	Pathology
[28]	Sivitz et al. (2014)	USA	Prospective	Pediatric	264	32.2	Fellow/Attending	Pathology/Follow-up
[29]	Corson-Knowles & Russell (2018)	USA	Prospective	Mixed	76	36.8	Attending/Fellow/Resident	Pathology/Follow-up
[30]	Nicole et al. (2018)	Canada	Prospective	Pediatric	117	43.6	Attending/Fellow	Pathology/Follow-up
[31]	Gungor et al. (2017)	Turkey	Prospective	Adult	264	64.0	Attending	Pathology/Follow-up
[32]	Abgottspon et al. (2022)	Switzerland	Retrospective	Adult (Pregnant)	61	49.2	Attending	Pathology/Clinical Course
[33]	Mustansar et al. (2024)	Pakistan	Prospective	Mixed	50	50.0	Attending	Pathology/Follow-up
[34]	Bourcier et al. (2018)	France	Prospective	Mixed	158	33.5	Attending	Pathology/Follow-up
[35]	Sharif et al. (2018)	Canada	Retrospective	Adult	90	26.7	Attending/Resident	Pathology/Follow-up
[36]	Ünlüer et al. (2016)	Turkey	Prospective	Adult	100	45.0	Attending	Pathology/Follow-up

Methodological Quality Assessment

**Figure 2:**
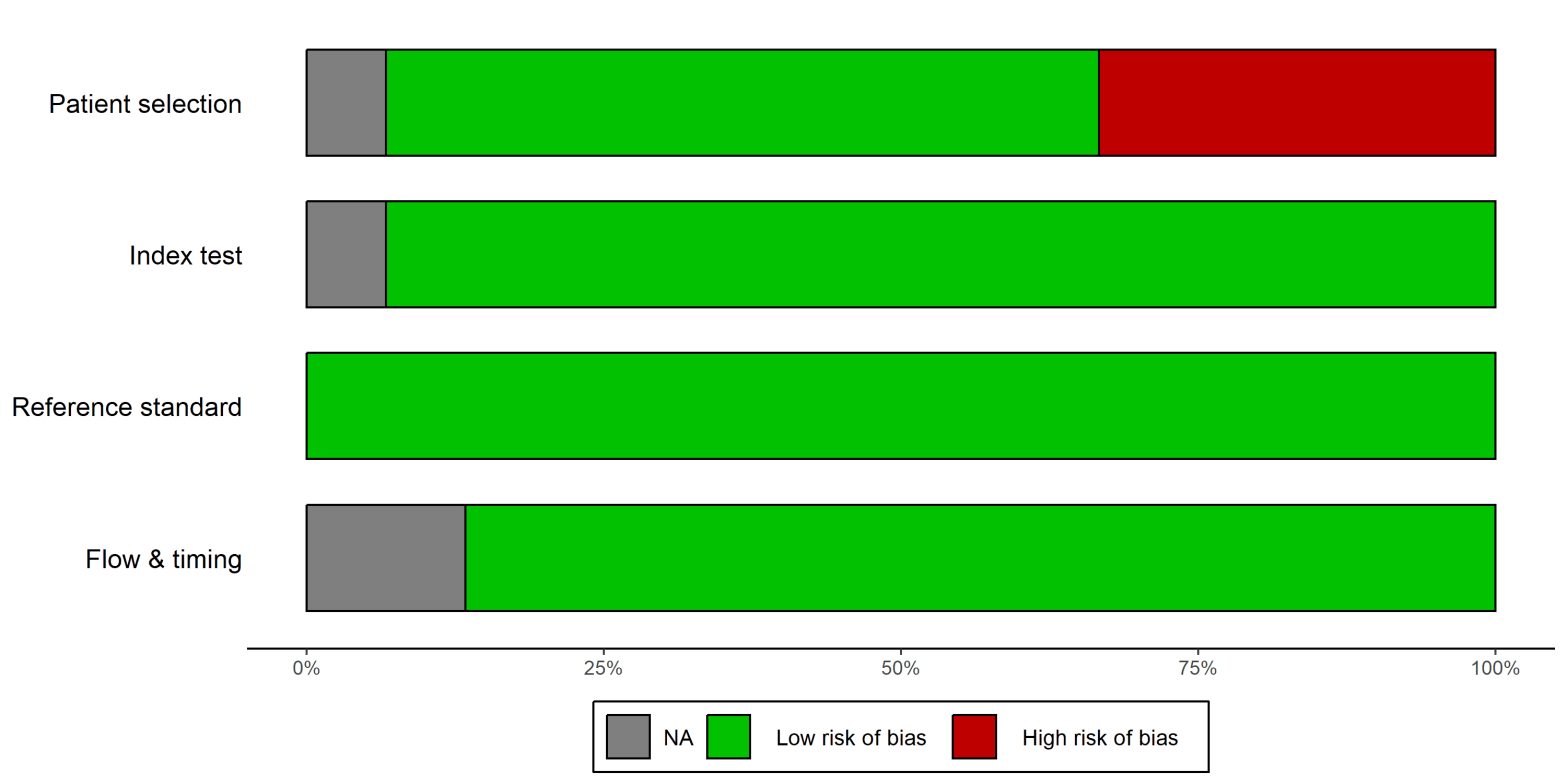
QUADAS-2 Risk of Bias Summary. Bar chart presenting the percentage of included studies [22-36] with Low, High, or Unclear risk of bias across the four domains: Patient Selection, Index Test, Reference Standard, and Flow & Timing. Abbreviations: QUADAS-2, Quality Assessment of Diagnostic Accuracy Studies-2 [11]

**Figure 3:**
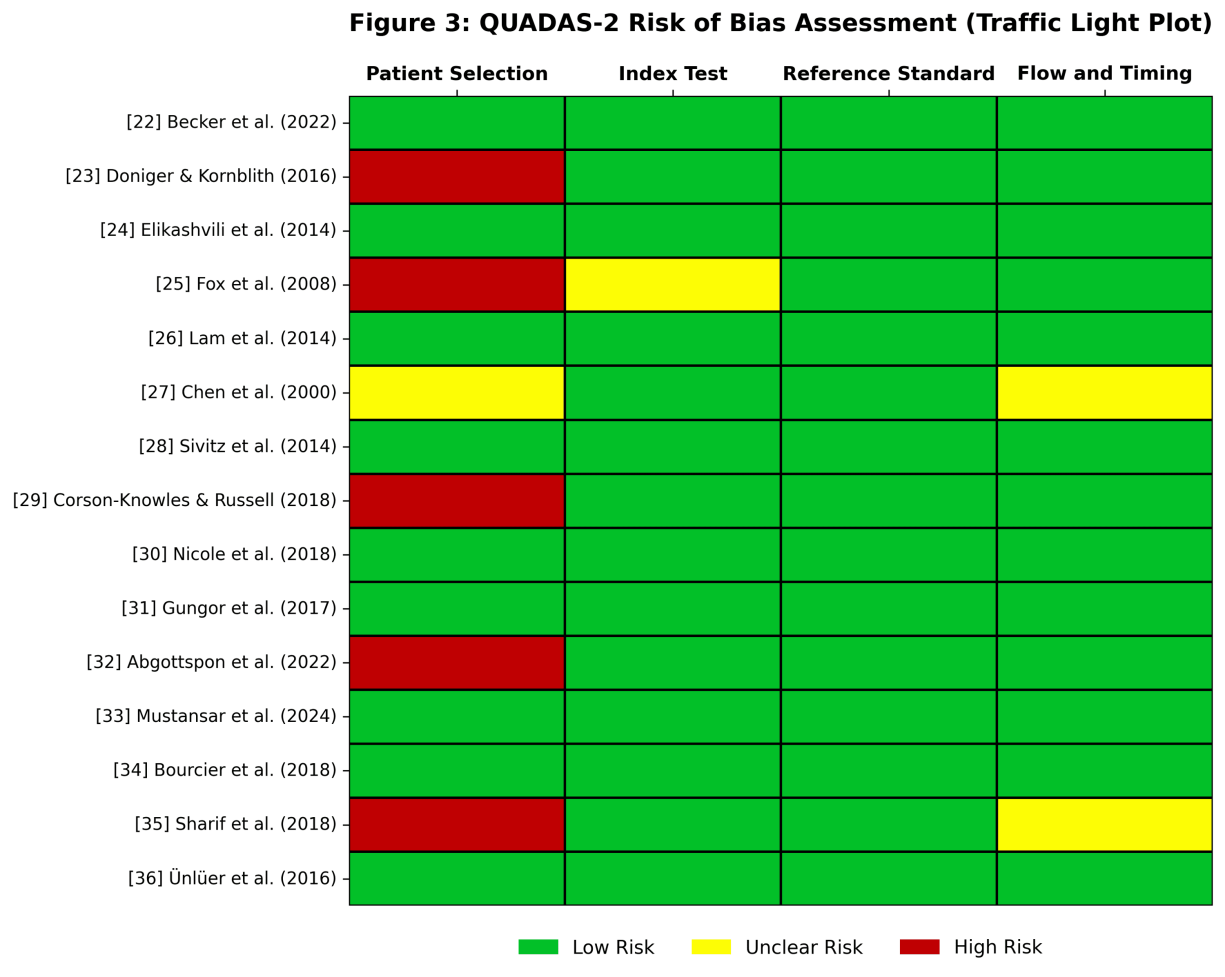
QUADAS-2 Traffic Light Plot. Detailed risk-of-bias assessment for each study included in the meta-analysis. Green indicates low risk, red indicates high risk, and gray indicates unclear risk [22-36]. Abbreviations: QUADAS-2, Quality Assessment of Diagnostic Accuracy Studies-2 [11]

The risk of bias and applicability concerns were evaluated using the QUADAS-2 tool [11]. A summary of the quality assessment is presented in Figure 2, with individual study details provided in Figure 3.

The patient selection domain exhibited the highest risk of bias. Six studies [23,25,29,32,35] were flagged as high risk because of the use of convenience sampling rather than consecutive enrolment, potentially introducing spectrum bias. Regarding the index test, most studies were rated as low risk, as POCUS was performed and interpreted prior to advanced imaging or surgical consultation. One study was rated unclear due to insufficient reporting of blinding [25]. All studies utilized appropriate reference standards (surgical pathology or clinical follow-up), resulting in a low risk of bias in this domain. Most studies maintained appropriate intervals between the index and reference tests, although two studies had unclear patient attrition or follow-up timing [27,35].

Diagnostic Accuracy

**Figure 4:**
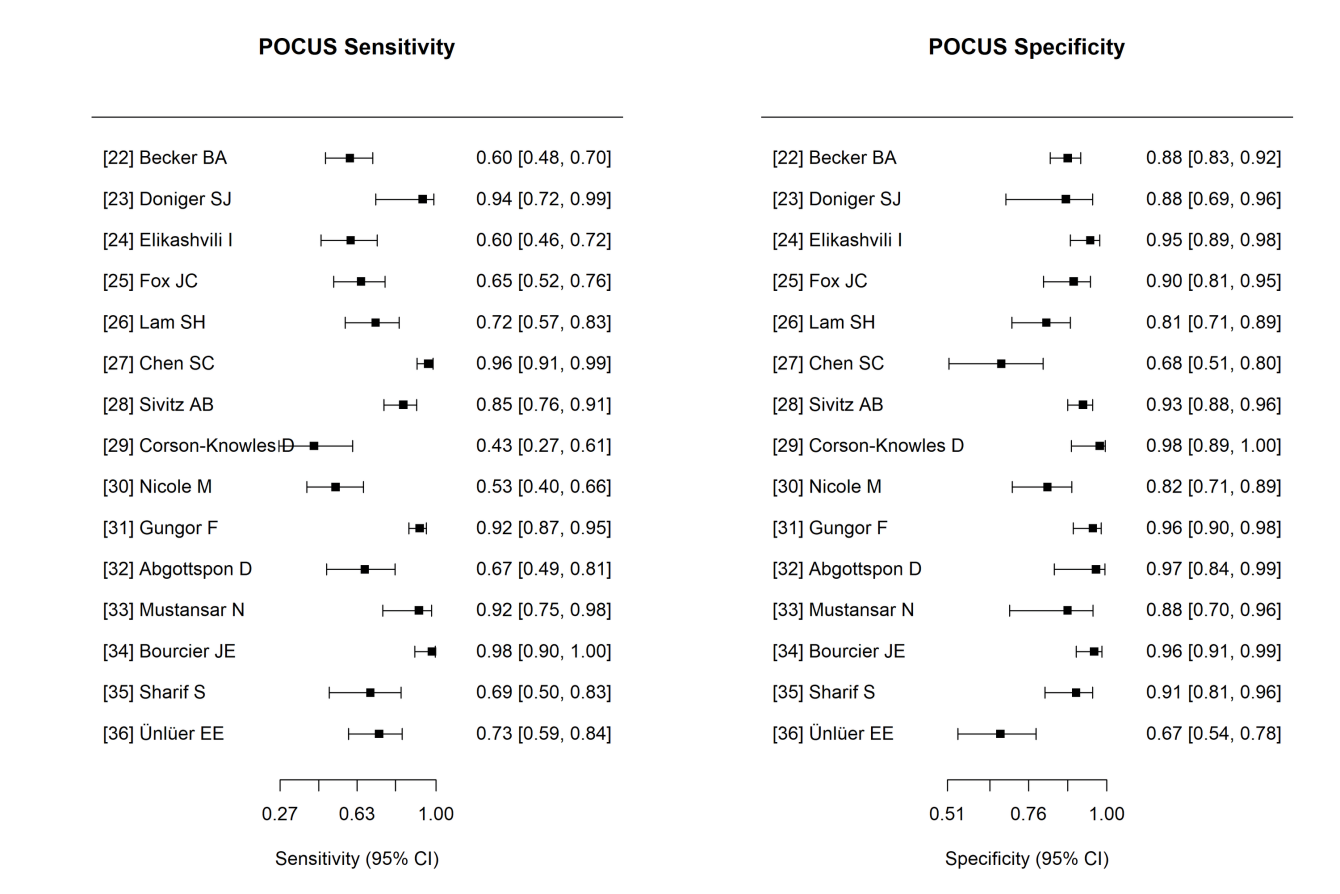
Coupled Forest Plots Displaying the Sensitivity and Specificity (with 95% Confidence Intervals) of POCUS for the Diagnosis of Acute Appendicitis in Each Included Study. Abbreviations: POCUS, point-of-care ultrasound

Forest plots of the sensitivity and specificity of the individual studies are displayed in Figure 4. There was substantial variability in sensitivity estimates, ranging from 43% [29] to 100% [26], whereas specificity remained consistently high across most studies, ranging from 67% [36] to 98% [29].

Application of the Reitsma bivariate random-effects model [14] yielded a combined sensitivity estimate of 0.78 (95% CI: 0.69-0.85) for POCUS in diagnosing acute appendicitis, while the combined specificity was found to be 0.89 (95% CI: 0.83-0.93). The pooled positive likelihood ratio (LR+) was 7.22 (95% CI: 4.51-11.54), and the pooled negative likelihood ratio (LR-) was 0.25 (95% CI: 0.17-0.36).

**Figure 5:**
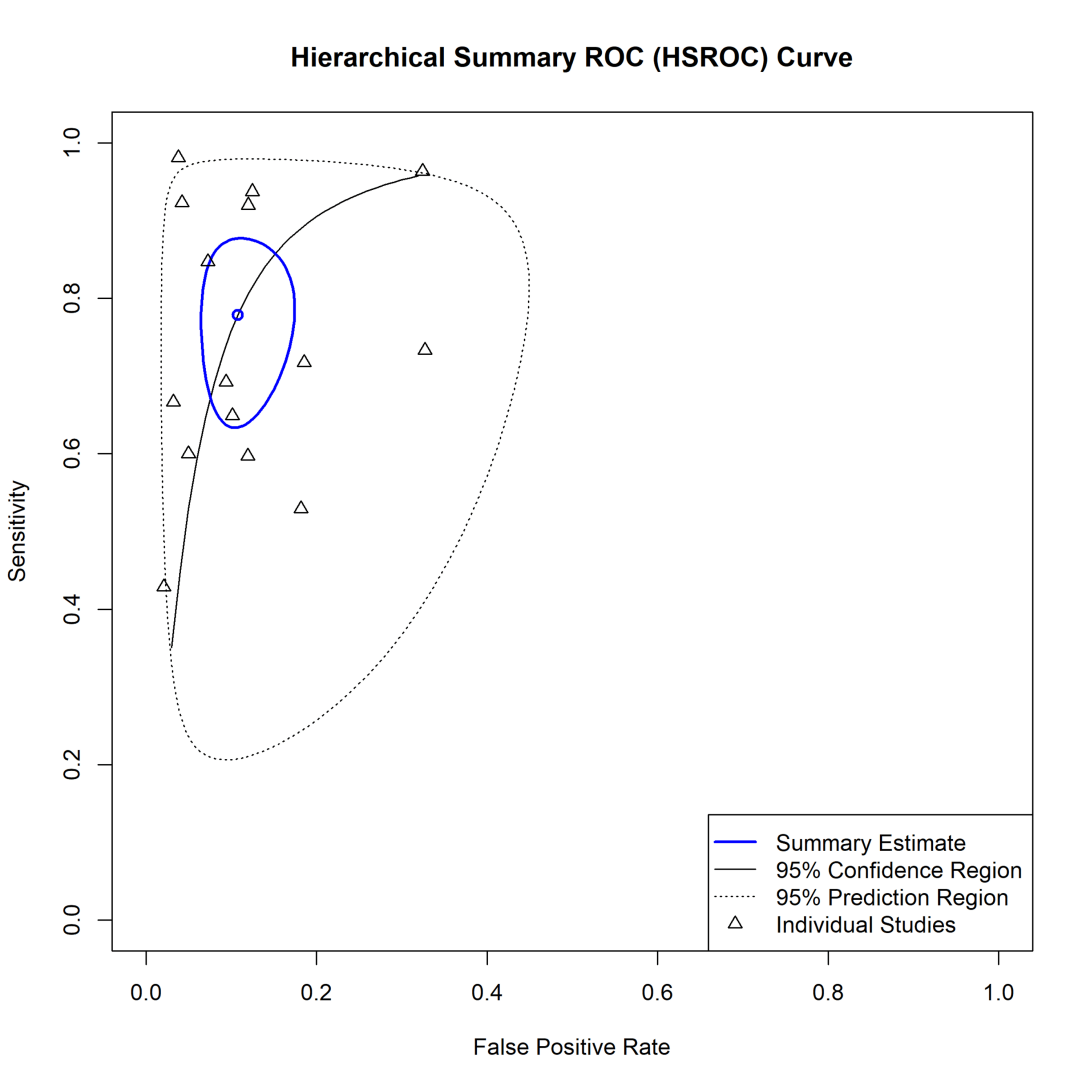
Hierarchical Summary Receiver Operating Characteristic (HSROC) Curve. The blue circle represents the summary sensitivity and specificity. The solid black line represents the 95% confidence region, and the dotted line represents the 95% prediction region. Data points represent individual studies included in the meta-analysis [22-36].

The Hierarchical Summary Receiver Operating Characteristic (HSROC) curve (Figure 5) visually depicts the trade-off between sensitivity and specificity. The summary point lies within a 95% confidence region (solid line), while the 95% prediction region (dotted line) is wide, indicating significant heterogeneity and anticipating variability in the performance of POCUS in future clinical settings.

Investigation of Heterogeneity

Statistical heterogeneity was high, with substantial inconsistency observed in both sensitivity and specificity measurements across the studies (I2 > 50%). Subgroup analysis via bivariate meta-regression indicated that Population Type (pediatric vs. adult/mixed) and Operator Experience significantly contributed to heterogeneity (P < 0.05). Studies utilizing fellowship-trained or highly experienced operators consistently reported higher sensitivity than those utilizing mixed-level residents or novice sonographers.

Inter-Rater Reliability

**Figure 6:**
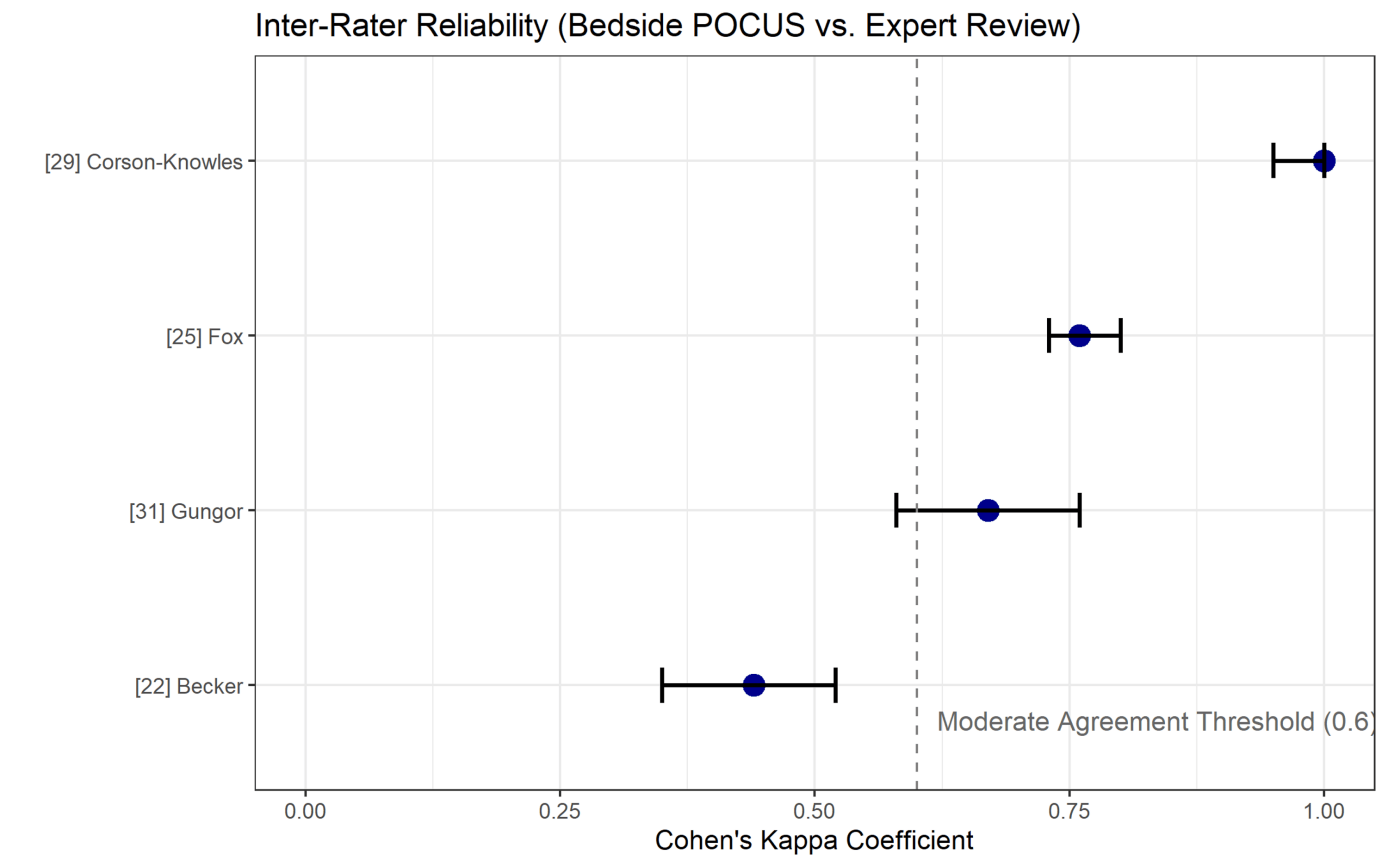
Inter-Rater Reliability Plot Summarizing Cohen’s Kappa Coefficients From Studies Comparing Bedside POCUS Interpretation With Expert Review. The dashed line indicates the threshold for moderate agreement (κ = 0.6). Abbreviations: POCUS, point-of-care ultrasound

Four studies reported inter-rater reliability data between bedside POCUS performed by emergency physicians and retrospective expert reviews [22,25,29,31]. Cohen’s kappa coefficients ranged from 0.44 to 1.0, with three studies demonstrating substantial to perfect agreement (kappa > 0.60). The variability in the agreement is summarized in Figure 6.

Publication Bias

**Figure 7:**
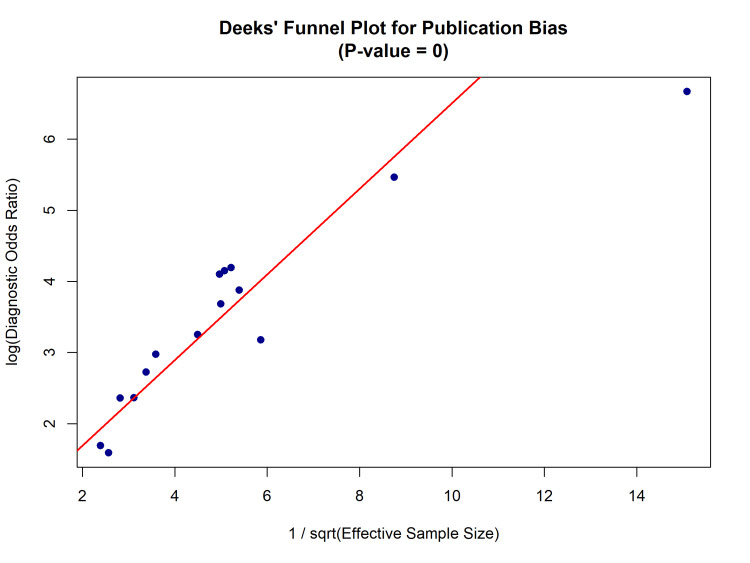
Deeks’ Funnel Plot for the Assessment of Publication Bias. Scatter plot assessing publication bias among the included studies [22-36]. The horizontal axis represents the log diagnostic odds ratio (DOR), and the vertical axis represents the inverse square root of the effective sample size​​​​​*.* Asymmetry (P < 0.01) indicates potential publication bias.

The Deeks’ funnel plot asymmetry test was performed to assess small-study effects and potential publication bias [20]. The visual inspection of the funnel plot (Figure 7) and the regression test yielded a P-value of < 0.01, indicating significant asymmetry, which suggests the presence of publication bias, likely due to the non-publication of smaller studies with negative or non-significant POCUS findings.

Clinical Utility

**Figure 8:**
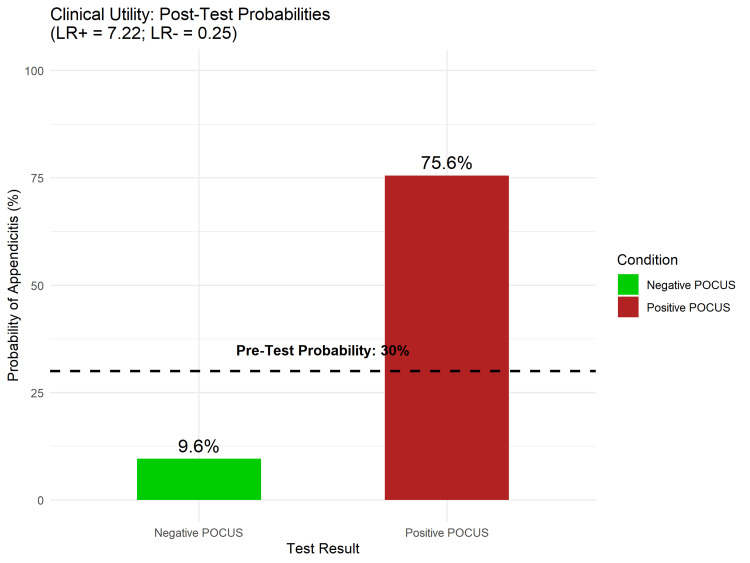
Fagan’s Nomogram Demonstrating the Clinical Utility of POCUS. Assuming a pre-test probability of 30%, a positive POCUS (Red Bar) raises the post-test probability to 76%, while a negative POCUS (Green Bar) lowers the probability to 10%. Pooled Likelihood Ratios (LR+ 7.22; LR- 0.25) derived from the bivariate model were used for calculation. Abbreviations: POCUS, point-of-care ultrasound

To illustrate the clinical application of these findings, a Fagan’s nomogram was generated (Figure 8) based on a hypothetical pre-test probability of 30%, which aligns with the prevalence of undifferentiated abdominal pain presentations [22]. A positive POCUS result increased the post-test probability of appendicitis from 30% to 76%, whereas a negative POCUS result decreased the post-test probability of appendicitis from 30% to 10%.

Certainty of Evidence

The certainty of the evidence was assessed using the GRADE approach (Grading of Recommendations Assessment, Development, and Evaluation). The overall certainty for the diagnostic accuracy of POCUS was graded as moderate. The evidence was downgraded due to serious risk of bias (attributed to convenience sampling in several included studies [23,25,29,32,35]), serious inconsistency (statistical heterogeneity with I^2^ > 50), and serious publication bias (asymmetry in Deeks’ funnel plot, P < 0.01). However, the rating was subsequently upgraded due to the large effect size (Pooled Positive Likelihood Ratio > 5) and the presence of a dose-response gradient, where higher diagnostic accuracy was observed in operators with advanced training compared to novices.

Discussion

This systematic review and meta-analysis synthesized data from 15 studies encompassing 2,021 patients to evaluate the diagnostic accuracy of POCUS performed by emergency physicians in suspected acute appendicitis. The findings indicate that POCUS has moderate sensitivity (78%) and high specificity (89%) for diagnosing appendicitis in the emergency department. While POCUS serves as a valuable tool for ruling in appendicitis, evidenced by a high positive likelihood ratio (LR+ 7.22), its utility as a standalone rule-out test is limited by a negative likelihood ratio (LR- 0.25), which is insufficient to definitively exclude pathology without further testing [14].

The pooled diagnostic accuracy reported in this study aligns with and, in some cases, exceeds that of previously published meta-analyses. Specifically, the observed specificity of 89% supports the conclusion that a positive POCUS scan, particularly when identifying a non-compressible appendix >6 mm, is highly reliable for confirming appendicitis [22,25]. This has significant clinical implications, as a definitive positive POCUS may obviate the need for computed tomography (CT), thereby reducing ionizing radiation exposure, costs, and time to surgical consultation [26,28]. The moderate sensitivity (78%) underscores that non-visualization of the appendix or an "indeterminate" scan should not reassure clinicians, necessitating secondary imaging such as formal ultrasound or CT [23,30].

Significant statistical heterogeneity was observed (I2 > 50), consistent with the operator-dependent nature of ultrasound [18]. The subgroup analysis revealed that operator experience is a critical moderator. Fellowship-trained and attending physicians consistently achieved higher diagnostic accuracy compared to residents or novice sonographers [22,31]. This dose-response relationship suggests that structured training and volume-based credentialing are essential for maximizing the utility of POCUS [24]. Furthermore, heterogeneity was driven by patient population, with pediatric studies generally showing higher specificity but variable sensitivity compared to adult cohorts, due to differences in body mass index (BMI) and anatomical visibility [28,30].

Strengths

The strength of this review is its robust methodological approach, which utilized the QUADAS-2 tool to assess the risk of bias [11] and the HSROC model to account for the threshold effect in diagnostic studies [15]. Furthermore, clinical utility was assessed using Fagan’s nomogram, which demonstrated that a positive POCUS result can increase the post-test probability of appendicitis from 30% to 76%, which is a clinically actionable threshold for surgical consultation [17].

Limitations

The significant publication bias identified by Deeks’ funnel plot asymmetry test suggests that smaller studies with negative or indeterminate findings may be underrepresented, potentially inflating pooled diagnostic estimates [20]. Additionally, the high risk of bias in patient selection (convenience sampling) across several included studies may limit the generalizability of the findings to all ED settings [25,29]. The inter-rater reliability analysis showed variability (Kappa 0.44-1.0), emphasizing that POCUS interpretation is subjective and benefits from expert over-read or quality assurance programs [16].

Clinical Implications and Future Directions

These results support the integration of POCUS into a stepwise diagnostic algorithm. For patients with suspected appendicitis, POCUS is the first-line imaging modality. A positive result enables rapid surgical referral, whereas an indeterminate or negative result mandates confirmatory imaging [23,26]. Future research should focus on standardized training curricula to reduce operator variability and prospective studies to validate POCUS-integrated clinical pathways, particularly in general community ED settings, where sonography expertise may vary [22,34].

## Conclusions

Point-of-care ultrasound performed by emergency physicians is a highly specific and moderately sensitive tool for diagnosing acute appendicitis. While it is effective for ruling in the disease, its moderate sensitivity and negative likelihood ratio mean it cannot independently rule out the diagnosis when the scan is negative or indeterminate. Its implementation can optimize resource utilization and reduce radiation exposure, provided that it is deployed by trained providers within a structured diagnostic pathway.
